# EBV-Positive Classic Hodgkin Lymphoma and Primary Nodal T-Cell/NK-Cell Lymphoma Arising in the Background of Follicular Lymphoma

**DOI:** 10.1155/2024/8810646

**Published:** 2024-09-10

**Authors:** Suravi Raychaudhuri, Zhao Ming Dong, Scott Knowles, Solomon Graf

**Affiliations:** ^1^ Fred Hutchinson Cancer Center, Seattle, WA, USA; ^2^ VA Puget Sound Health Care System, Seattle, WA, USA; ^3^ University of Washington, Seattle, WA, USA

## Abstract

EBV-positive primary nodal T-cell/NK cell lymphoma (TNKL) is a rare diagnosis with a poor prognosis. No relationship with follicular lymphoma (FL), classic Hodgkin lymphoma (cHL), or other non-Hodgkin lymphomas is established. We describe a case of Epstein–Barr virus (EBV)-positive cHL and EBV-positive primary nodal TNKL in the background of an antecedent FL, with all 3 subtypes identified in a single lymph node biopsy from an immunocompetent patient. Intensive frontline therapy achieved only a temporary response, with subsequent rapid progression associated with hemophagocytic lymphohistiocytosis (HLH). We discuss the relationship of the three lymphoma subtypes and the potential roles of EBV and immune dysregulation as contributing factors to this previously undescribed composite lymphoma.

## 1. Introduction

EBV-associated natural killer (NK) and T-cell lymphomas, including extranodal NK/T-cell lymphoma (previously termed “nasal type”) (ENKL) and EBV-positive primary nodal TNKL, are very aggressive lymphomas more common in Asian and South American populations [1, 2]. Presentation typically includes widespread lymphadenopathy with B-symptoms; prognosis is poor, with median survival of 6 months or less and no established standard therapy [3, 4]. EBV-positive primary nodal TNKL is generally of cytotoxic T-cell rather than NK cell origin with frequent mutations in the epigenetic-modifier genes TET2 and DNMT3A [5]. Underlying immune deficiency or impaired immune responses is present in most cases [5].

## 2. Case Presentation

A 68-year-old White man without significant past medical history was diagnosed with FL with skin involvement. He remained asymptomatic for 3 years before presenting with abdominal pain and night sweats. 18F-fluorodeoxyglucose (FDG) positron emission tomography/computed tomography (PET/CT) showed bulky lesions in the iliac and inguinal regions (maximum SUV = 58.5) concerning for histologic transformation.

**Figure 1:**
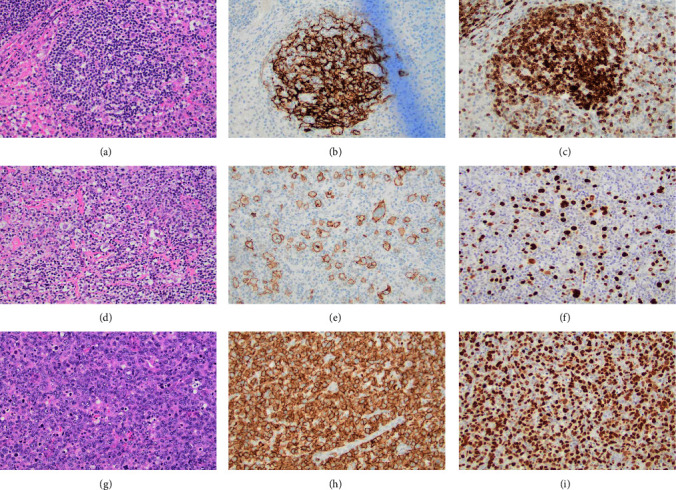
Composite lymphoma with co-existence of classic (grade 1 to 2) follicular lymphoma (a–c), EBV-positive classic Hodgkin lymphoma (d–f), and EBV-positive nodal T- and NK-cell lymphoma (g–i) in a left groin lymph node. (a) Section shows a neoplastic follicle composed of predominantly centrocytes (H&E, X20). (b) The neoplastic follicle contains dendritic cell meshworks as confirmed by IHC (CD21, X20). (c) Follicular cells are positive for BCL-2 as demonstrated by IHC (BCL-2, X20). (d) Section shows large Hodgkin/Reed-Sternberg-like cells and variants (H&E, X20). (e) The neoplastic cells are positive for CD30 as demonstrated by IHC (CD30, X20). (f) The neoplastic cells contain EBV as demonstrated by EBER in situ study (EBER in situ, X20). (g) Section shows a diffuse infiltration of large atypical lymphoid cells (H&E, X20). (h) The neoplastic cells are positive for CD8 as demonstrated by IHC (CD8, X20). (i) The neoplastic cells contain EBV as demonstrated by EBER in situ study (EBER in situ, X20). H&E, Hematoxylin and Eosin. IHC, immunohistochemical stain.

Inguinal lymph node excisional biopsy showed a composite lymphoma comprised of clonally distinct EBV-positive cHL, nodular sclerosis type (occupying 40–50% of lymph node space), EBV-positive primary nodal TNKL (50–60%), and classic (grade 1-2) FL (1-2%) (Figure 1). In the cHL component, IHC was positive for CD30, CD15, CD20 (weak), c-MYC, BCL6, and BCL2. In the TNKL component, tumor cells were positive by IHC for CD3, CD8, and TCR beta. Cells were negative for CD30, CD15, and BCL2. EBV in situ hybridization was strongly positive in both cHL and TNKL components. Clonality studies were performed according to standard techniques on the original FL specimen and the excised lymph node after cell sorting. B-cell clonality was assessed using PCR for rearrangement of the IgH, IgK, and Ig lambda genes. B-cell clonality of the antecedent FL was shared with the FL component in the excised lymph node but distinct from the cHL. T-cell clonality was assessed via T-cell receptor (TCR) gamma gene rearrangement PCR. Clonal TCR rearrangement was identified in the EBV + nodal NKTL but not in the FL population or the cHL. Thus, no clonal relationship was found between the three lymphoma types based on TCR and IgH rearrangement studies.

Next generation sequencing on paraffin embedded blocks identified two TET2 mutations (p.A1508Lfs∗63, variant allele frequency (VAF) 11% and p.Q321∗, VAF 3%) and an ASXL1 mutation (p.G645Vfs∗58, VAF 5%). The subsequent biopsy showed the same 2 TET2 mutations (VAF 28% and 17%, respectively) without accumulation of further mutations. These TET2 and ASXL1 mutations are likely age-related clonal hematopoiesis of indeterminate potential and are not considered clinically significant.

The patient was hospitalized with hypoxic respiratory failure due to lung involvement by lymphoma and treated with 2 cycles of cyclophosphamide, doxorubicin, vincristine, and prednisone (CHOP); pembrolizumab was added to the subsequent 4 cycles of CHOP given its potential activity in EBV-associated extranodal T/NK cell lymphoma and established role in cHL [6, 7]. Early disease response was achieved with normalization of the patient's respiratory status and decrease in serum EBV from 380,000 IU/mL to 120 IU/mL; interim PET/CT showed partial response. Approximately 1 month after completing the course of induction therapy, the patient presented with refractory distributive shock, multiorgan failure, and serum ferritin of 68,000 ng/mL. The patient elected to pursue comfort-focused management and died shortly thereafter. HLH associated with active T-cell lymphoma was shown on autopsy.

## 3. Discussion

To our knowledge, this is the first report of a composite EBV-positive cHL and primary nodal TNKL arising in the background of FL in an immunocompetent patient. EBV-positive primary nodal TNKL is a rare diagnosis with a poor prognosis, typically found in East Asian, elderly, and/or immunocompromised individuals. There is no known relationship with the development of primary nodal TNKL and cHL or other non-Hodgkin lymphomas [2].

EBV seropositivity is nearly ubiquitous in adults across the globe. The relationship between EBV infection and lymphomagenesis is complex, and as recently reviewed by Bednarska et al. [8], most lymphomas driven by EBV infection are of B-cell origin; EBV-associated Burkitt lymphoma, diffuse large B-cell lymphoma, and Hodgkin lymphoma are the most common. Approximately 10 percent of EBV-associated lymphomas are of T- or NK- cell type though the mechanism behind EBV oncogenesis in these entities unclear. EBV latency in naïve B cells is thought to promote both cHL and extranodal TNKL through expression of viral genes including EBNA-1, EBNA-LP, latent membrane protein-1, -2A, and -2B, EBERs, and BARTs, comprising the so-called EBV latency type II gene pattern; separate sets of EBV genes and distinct latency types are associated with other lymphomas [9].

A fascinating aspect of this case is the absence of clonal relationship shown between the lymphoma subtypes identified within a single excised lymph node. The strongly positive EBV presence in both the cHL and TNKL in conjunction with the very elevated serum EBV DNA level at diagnosis support a linked origin and causal role for EBV infection. One hypothesis holds that the antecedent FL promoted an immunosuppressed microenvironment through recruitment of regulatory T cells and secretion of immune-dampening signals such as transforming growth factor-*β* resulting in unchecked lymphomagenesis supported by EBV infection [10]. In addition to EBV's role in certain aggressive B-cell non-Hodgkin lymphomas, case reports of FL transformation to cHL have also implicated EBV [11].

This case illustrates the important interplay of the immune system with lymphoma, highlighting the potential for immune dysregulation associated with lymphoma to complicate the disease biology and treatment. Efforts investigating EBV-directed treatments, including antivirals and adoptive cell therapy, are ongoing and results eagerly awaited [12].

## Data Availability

The clinical and pathologic data used to support the findings of this study are included within the article.
